# Carbon supported Ag nanoparticles as high performance cathode catalyst for H_2_/O_2_ anion exchange membrane fuel cell

**DOI:** 10.3389/fchem.2013.00016

**Published:** 2013-09-23

**Authors:** Le Xin, Zhiyong Zhang, Zhichao Wang, Ji Qi, Wenzhen Li

**Affiliations:** Department of Chemical Engineering, Michigan Technological UniversityHoughton, MI, USA

**Keywords:** non-platinum catalyst, electrocatalyst, nanoparticles, anion exchange membrane, fuel cell, oxygen reduction

## Abstract

A solution phase-based nanocapsule method was successfully developed to synthesize non-platinum metal catalyst—carbon supported Ag nanoparticles (Ag/C). XRD patterns and TEM image show Ag nanoparticles with a small average size (5.4 nm) and narrow size distribution (2–9 nm) are uniformly dispersed on the carbon black Vulcan XC-72 support. The intrinsic activity and pathway of oxygen reduction reaction (ORR) on the Ag/C and commercial Pt/C were investigated using rotating ring disk electrode (RRDE) tests at room temperature. The results confirmed that the 4-electron pathway of ORR proceeds on small Ag nanoparticles, and showed comparable ORR activities on the self-prepared Ag/C and a commercial Pt/C. A single H_2_-O_2_ anion exchange membrane fuel cell (AEMFC) with the Ag/C cathode catalyst exhibited an open circuit potential of 0.98 V and a peak power density of 190 mW/cm^2^ at 80°C.

## Introduction

H_2_-based proton exchange membrane fuel cells (PEMFCs) have been extensively studied in recent decades as an alternative power source, due to their unique advantages of high energy conversion efficiency and zero emission (Vielstich et al., 2003). However, in order to improve the kinetics of oxygen reduction reaction (ORR) and maintain long catalyst lifetime, Pt-group metals (PGMs) have to be employed as cathode catalysts in PEMFCs (Gasteiger et al., 2005). In high pH media, the ORR kinetics can be greatly improved due to enhanced ion transport and facile charge transfer (Spendelow and Wieckowski, 2007). Recently, low-temperature anion exchange membrane fuel cells (AEMFCs) have re-surged after decades due to introduction of novel solid anion exchange membranes that have demonstrated high anion conductivity and chemical stability, (Varcoe and Slade, 2005; Varcoe et al., 2006a,b; Lu et al., 2008; Gu et al., 2009; Li et al., 2011). An attractive merit of AEMFC is inexpensive PGMs can be used as electrocatalyst (Varcoe et al., 2006b; Spendelow and Wieckowski, 2007; Lu et al., 2008; Li et al., 2011). Among all non-PGM catalysts, Ag has exhibited very high ORR intrinsic activity and durability in high pH electrolyte (Spendelow and Wieckowski, 2007; Antolini and Gozalez, 2009). In addition, the price of Ag is about 20 US$/oz, which is about 75 times lower than precious metal Pt, 1500 US$/oz (Nasdaq Precious Metal Online Price). Carbon supported Ag electrocatalysts have aroused extensive interests as an alternative to Pt for ORR in alkaline media (Furuya and Aikawa, 2002; Okajima et al., 2005; Chatenet et al., 2006; Varcoe et al., 2006b; Spendelow and Wieckowski, 2007; Lu et al., 2008; Antolini and Gozalez, 2009). Blizanac and co-workers have demonstrated that on Ag (111) single crystal, the ORR proceeds via 4-electron pathway in high alkaline media (i.e., pH >15), with very little production of undesirable H_2_O_2_ by-product. It was also suggested that though Ag-O_ad_ interaction is weaker when compared with Pt, it is still strong enough to facilitate dissociation of the O-O bond (Blizanac et al., 2007). Consideration was also given to the effect of carbon support on the pathway of ORR in alkali (Lima et al., 2006). 4-electron pathway was confirmed with regard to carbon supported large Ag particle (i.e., >20 nm) electrocatalysts (Meng et al., 2006). Ag nanowire catalysts were successfully synthesized and it was concluded that 4-electron ORR was predominant on the Ag nanostructures (Kostowskyj et al., 2010). The Ag/C with high loading of 4 mg_Ag_/cm^2^ has been tested in the single AEMFC as cathode catalyst and a peak power density of *ca*. 47 mW/cm^2^ was obtained at 50°C (Varcoe et al., 2006b). Park group reported a peak power density of 30 mW/cm^2^ when using 2 mg_Ag_/cm^2^ Ag/C (40 wt%) (Park et al., 2008). Currently, it is in high demand to study small Ag nanoparticles and increase the output power density of H_2_/O_2_ AEMFCs.

Recently, solution phase-based synthesis methods have been emerging as one of the most promising approaches to accurately control the size, shape and structure of metallic nanoparticles, which could serve as promising oxygen reduction electrocatalysts (Chen et al., 2007; Lim et al., 2009; Mazumder et al., 2010; Peng et al., 2010). In our lab, we successfully developed a convenient solution-phase nanocapsule method to prepare Pt-Co nanoparticles (Li et al., 2010), PtNi@Pt core-shell nanoparticles (Li and Haldar, 2010), PdFe nanorods (Li and Haldar, 2009), and Pd nanoleaves (Zhang et al., 2011a) electrocatalysts and obtained improved electrocatalytic activity to ORR in both acid and alkaline electrolytes, due to their very small diameter of <5 nm (thus offer large catalytic active surface area) (Li and Haldar, 2010; Li et al., 2010), optimized crystalline structure (Li and Haldar, 2009; Zhang et al., 2011a,b) and tuned electronic properties (Li and Haldar, 2009; Zhang et al., 2011a,b).

In this article, we modified this nanocapsule method to prepare carbon supported non-PGMs Ag nanoparticles (with average size of 5.4 nm), at near room temperature. The physical characterizations were conducted by XRD, TEM, and ICP-AES. The comparison of ORR mechanisms and performances between Ag/C and commercial Pt/C electrocatalysts was investigated in both half cell and single AEMFC. Our preliminary results showed one of the highest single AEMFC performance with Ag/C cathode catalyst among all previously reported results, demonstrating that Ag/C is a promising cathode catalyst substitute to Pt/C for H_2_/O_2_ AEMFCs.

## Experimental section

**Scheme 1:**
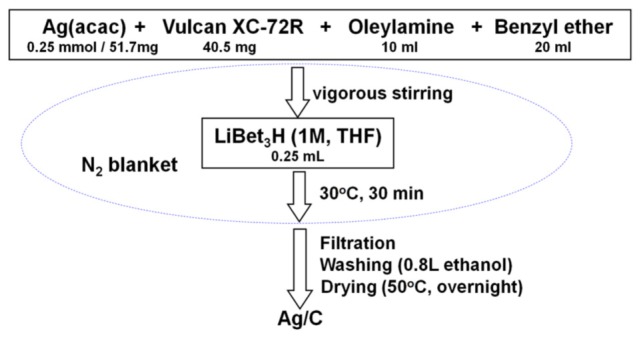


The Ag/C catalyst with a metal loading of ~40% was synthesized through a modified solution phase-based nanocapsule method (Li and Haldar, 2009, 2010; Li et al., 2010; Mazumder et al., 2010; Zhang et al., 2011a,b). Briefly, 51.7 mg Ag(acac) (0.25 mmol) and 40.5 mg Vulcan XC-72R carbon black were mixed in 10 ml oleylamine and 20 ml benzyl ether by vigorous stirring under a N_2_ blanket. The temperature was kept at 30°C, while 0.25 ml LiBEt_3_H (1.0 M THF solution) was injected into the solution. After held at that temperature for an additional 30 min, the final product Ag/C was collected after filtration, washed with 800 ml ethanol, and drying overnight in a vacuum oven. The synthesis procedure is illustrated in Scheme 1.

The morphology, structure, and metal loading of Ag/C catalysts were analyzed by X-ray diffraction (XRD, Scintag XDS-2000, Cu K_α_ radiation, λ = 1.5406 Å), transmission electron microscopy (TEM, JEOL 2010, 200 kV) and inductively coupled plasma atomic emission spectroscopy (ICP-AES).

A conventional three-electrode-cell setup (electrolyte cell AFCELL3, Pine) consisting of a glassy carbon disk (0.1963 cm^2^)/Pt ring (0.1099 cm^2^) working electrode, a Hg/HgO /1.0 M KOH reference electrode and a Pt wire counter electrode, was used for rotating ring disk electrode (RRDE) tests of the Ag/C and Pt/C (40 wt%, BASF-FuelCell) catalysts. Before testing, 1.0 mg catalyst was dispersed in 1.0 ml isopropanol by sonication to form a uniform ink. The working electrode was prepared by depositing 20 μL of the ink on the glassy carbon electrode and left to dry at room temperature. Next, 10 μL of 0.05 wt% AS4 (Tokuyama) ionomer solution was drop-casted on the catalyst layer in order to attach the electrocatalyst particles on the glass carbon substrate. The RRDE test was conducted in O_2_-saturated 0.1 M KOH electrolyte. Linear scan voltammetry at a sweep rate of 10 mV/s was performed from −0.9 to 0.1 and 0.2 V at room temperature, on Ag/C and Pt/C, respectively. The working electrode rotation rate is 2500 rpm. The Pt ring electrode was potentiostated at 0.5 V, with the collection efficiency *N* = 0.23 that is determined by using the typical compound, K_3_Fe(CN)_6_ (Paulus et al., 2001).

The single AEMFC test was performed on a fuel cell test system (Scribner 850e). The single fuel cell stack consists a pair of graphite blocks with serpentine flow pattern and copper-plated current collectors (Fuel Cell Technology). The catalyst ink was prepared by mixing carbon supported catalyst, AS4 (5wt%, Tokuyama) anion exchange ionomer and 1-propanol. Subsequently, the catalyst ink was sprayed directly onto both sides of the anion exchange membrane (A201, Tokuyama, 28 μm) until the desired catalyst loadings (0.2 mg_Pt_/cm^2^ for the anode and 1.0 mg_Ag_/cm^2^ for the cathode) were obtained. For comparison, the cathode incorporated with a Pt loading of 0.5 mg_Pt_/cm^2^ (Pt/C, 40 wt%, BASF-Fuel Cell) was also prepared. The MEA was fabricated by mechanically sandwiched the anode gas diffusion layer (SGL Carbon 25CC), catalyst-coated anion exchange membrane and cathode gas diffusion layer (carbon paper, TGP-H-060, Toray) into the single fuel cell stack. The MEA has an area of 5 cm^2^. High purity H_2_ and O_2_ (99.999%) were fed into anode and cathode with a constant flow rate of 200 ml/min and a back pressure of 30 psi. The cell temperature was kept at 80°C, and the relative humidity for both anode and cathode is 100%. The MEA was activated at 0.1 V until the current density got stable. The I-V polarization curve was obtained by applying a constant voltage and collecting corresponding current density.

## Results and discussion

**Figure 1:**
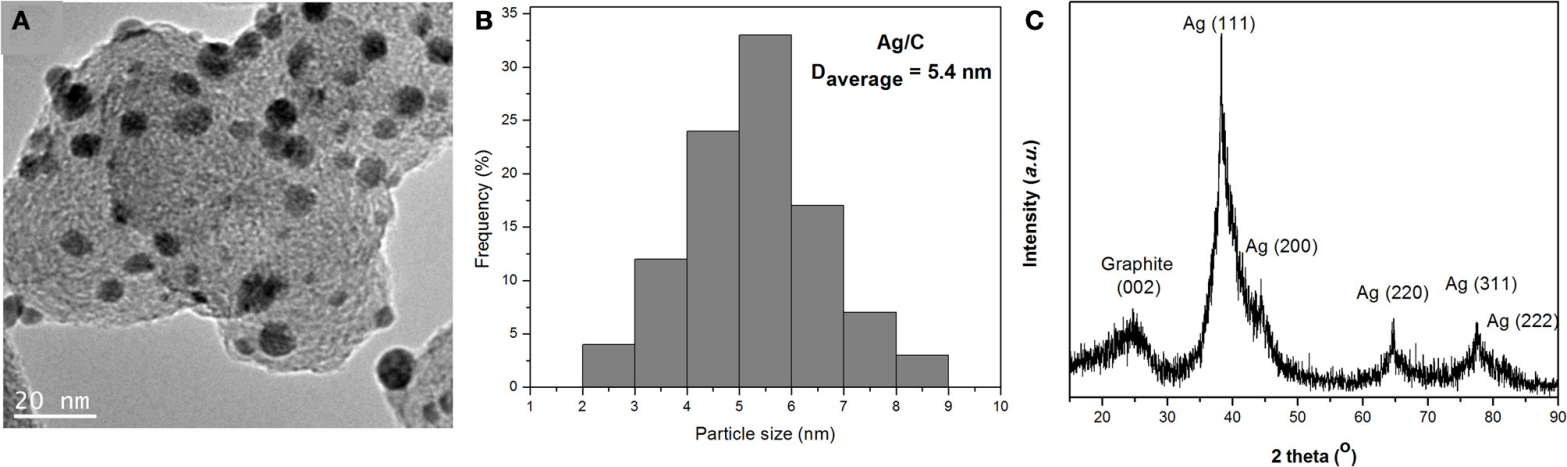
**TEM image (A), particle size histogram (B) and XRD patterns (C) of Ag/C catalyst**.

A typical TEM image of Ag/C catalyst is shown in Figure 1A. Uniformly dispersed Ag nanoparticles were observed on carbon black support. The corresponding particle size histogram in Figure 1B evaluated from 100 random particles in an arbitrarily chosen area presents a narrow distribution of 2–9 nm, centered at 5.4 nm for Ag nanoparticles, which indicates the modified nanocapsule method has a strong ability to control over nanoparticle size and morphology. The XRD patterns in Figure 1C show a typical Ag face centered cubic (fcc) sturcture, with the peaks at 38.2, 44.3, 64.4, 77.5, and 81.5° assigned to Ag (111), (200), (220), (311), and (222) facets, respectively. The average particle size calculated from the Ag (220) diffraction peak by Debye-Sherrer equation is 4.2 nm, which confirms the small size of Ag particles prepared by this nanocapsule method. The metal loading of Ag/C catalyst has been determined by ICP-AES to be 31%. The lower metal loading (31% *vs*. setting value of 40%) may be due to the small amount of surfactant residue still left on the carbon supported Ag catalyst after the filtration process.

**Figure 2:**
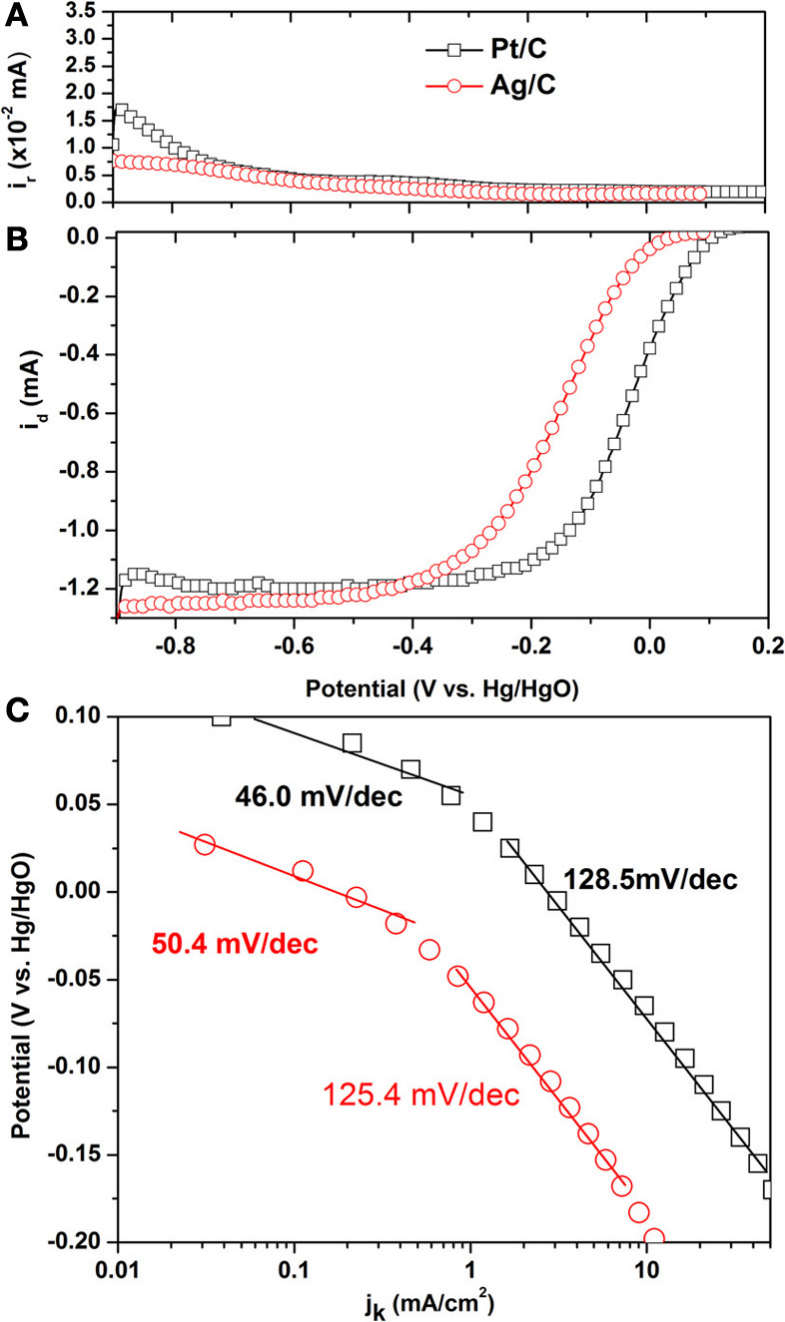
**Linear sweep voltammograms of Pt/C and Ag/C catalysts for the oxygen reduction reaction in O_2_-saturated 0.1 M KOH.** Scan rate: 10 mV/s; rotation rate: 2500 rpm; ring potential: 0.5 V; collection efficiency: 0.23; room temperature. **(A)** Ring current; **(B)** Steady state polarization curves; **(C)** Tafel plots.

Steady state polarization curves for the ORR on Ag/C and its ring current corresponding to HO_2_^−^ oxidation on Pt ring electrode are shown in Figures 2A,B and compared with those of the commercial Pt/C. The onset potential on Ag/C is 0.034 V, which is lower than that of Pt/C (0.104 V). It is interesting to observe that the ring current on Ag/C is lower than that of Pt/C, indicating a lower HO_2_^−^ generation on Ag/C. The HO_2_^−^ is the main ORR by-product that not only reduces the energy efficiency by 50%, but also could deteriorate the ionomer and membrane. The lower HO_2_^−^ generation rate suggests Ag/C is an efficient and safe catalyst when employed in AEMFCs. The number of transferred electron (*n*) during ORR was calculated according to equation 1 (Paulus et al., 2001):

(1) $$n=\frac{4i_{d}}{i_{d}+\left(\frac{i_{r}}{N}\right)}$$

where *i*_d_ is the disk current, *i*_r_ is the ring current and *N* = 0.23 is the RRDE collection efficiency. The calculated *n* for Ag/C and Pt/C are 3.942 and 3.917, respectively. This indicates that both of them catalyze the ORR mainly through the four-electron pathway in alkaline electrolyte. It has been reported that on Ag (111) single crystal, the oxygen reduction proceeds 4-electron pathway in base, while 2-electron pathway in acid, suggesting that although Ag-O_ads_ interaction is weaker than Pt-O_ads_, but it is still strong enough to break the O-O bond in alkaline electrolyte. While in acid electrolyte, anion coverage is relatively high, thus disabling the surface to provide the required number of virgin sites for adsorption of O_2_ and subsequent O-O bond cleavage processes (Blizanac et al., 2007).

Figure 2C shows the Tafel plots on Ag/C and Pt/C, in order to compare their intrinsic activity. The kinetic current density *j*_k_ is estimated by correcting the mass transport through the equation 2, which is derived from Levich-Koutecky equation by assuming that O_2_ reduction obeys first-order kinetics (Gojkovic et al., 1999).

(2) $$j_{k}=\frac{j.j_{1}}{j_{1}-j}$$

where *j*_1_ is the diffusion limiting current density and *j* is the collected current density. In Figure 2C, both of the Tafel slopes on Ag/C and Pt/C can be divided into two regions. The value of 50.4 and 46.0 mV/decade is for Ag/C and Pt/C in the low overpotential region, which could be attributed to the transfer of the first electron as a rate determine step and Temkin condition of intermediate adsorption (Sepa et al., 1981). The Tafel slope of 125.4 and 128.5 mV/decade is obtained for Ag/C and Pt/C in the high overpotential region. The close values of Tafel slopes of Ag/C and Pt/C also suggest the ORR pathway and rate determine step occurs similarly on the two catalysts.

**Figure 3:**
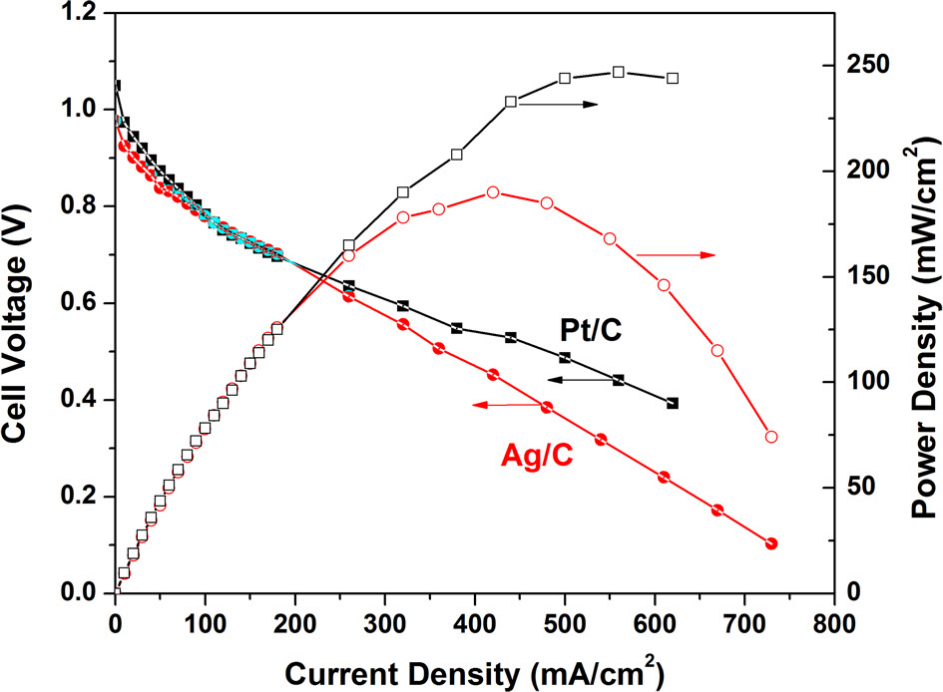
**Polarization and power density curves of anion exchange membrane fuel cells.** Anode: Pt/C, 0.2 mg_Pt_/cm^2^; cathode: Ag/C (self-prepared, 1.0 mg_Ag_/cm^2^), or Pt/C (BASF-Fuel Cell, 0.5 mg_Pt_/cm^2^); membrane: A201 (Tokuyama); cell temperature: 80°C; relative humidity of anode and cathode: 100%; flow rate of H_2_ and O_2_: 200 ml/min; backpressure of anode and cathode: 30 psi.

The polarization and power density curves of the H_2_/O_2_ AEMFCs with Ag/C and Pt/C cathode catalysts are shown in Figure 3. At an operation temperature of 80°C, the open circuit voltages of Pt/C and Ag/C-based AEMFCs are 1.05 and 0.98 V, respectively, which is in good agreement with the trend of the onset potential in linear sweep voltammograms. The maximum power densities can reach up to 247 and 190 mW/cm^2^ for Pt/C and Ag/C, respectively. When the current density is less than 100 mA/cm^2^ (kinetics-controlled region), the performance of Ag/C is slightly lower than Pt/C. However, in the internal resistance-controlled region (100–600 mA/cm^2^), the AEMFC employing Ag/C cathode catalysts showed an internal resistances of 0.177 Ωcm^2^, which is lower than the one with Pt/C cathode catalyst (0.235 Ωcm^2^). The competitive AEMFC performance with Ag/C catalyst can be explained as that the high pH level environment favors ORR kinetics on Ag/C, but inhibits ORR kinetics on Pt/C, which is attributed to different extents of surface oxidation on Ag and Pt. Unlike Pt, Ag has a filled d band that is much less oxophilic than Pt. Therefore, surface oxidation of Ag with the increasing of local alkaline concentration is not a significant factor in determining ORR activity (Blizanac et al., 2007; Spendelow and Wieckowski, 2007). Recent work from Yan lab reported AEMFCs with peak power density of 178 and 250 mW/cm^2^ based on the FAA and TPQPOH152 anion exchange membranes at 70°C, respectively (Gu et al., 2009). Popov group has also shown an AEMFC with a peak power density of 196 mW/cm^2^ using Pt cathode catalyst and A201 membrane (Tokuyama) at 50°C (Li et al., 2011). Our performance baseline of the AEMFC with Pt/C catalyst (247 mW/cm^2^ at 80°C) is close to these recent-published work. The small Ag nanoparticle catalyst has demonstrated a very promising initial catalytic activity of ORR, and the studies of its long-term stability and the overall AEMFC durability are underway in our group.

## Conclusion

In summary, a solution phase-based nanocapsule method has been developed to prepare Ag/C catalyst. The characterizations reveal that Ag nanoparticles have a small size of 5.4 nm and narrow size distribution of 2–9 nm. High activity and 4-electron reaction pathway of ORR in alkaline media on Ag/C have been confirmed by using RRDE tests. The H_2_-O_2_ AEMFCs with Ag/C and Pt/C cathode catalysts show comparable performances: peak power density of 190 mW/cm^2^ for Ag/C and 247 mW/cm^2^ for Pt/C at 80°C, which suggests Ag/C is a competitive substitute to Pt/C as AEMFC cathode catalyst.

### Conflict of interest statement

The authors declare that the research was conducted in the absence of any commercial or financial relationships that could be construed as a potential conflict of interest.
